# Grafting of Zwitterionic Polymers on Zirconia Surfaces: An XPS Investigation

**DOI:** 10.3390/ma18102279

**Published:** 2025-05-14

**Authors:** Clément Dezanet, Diana Dragoe, Arnaud Fouchet, Jérôme Lecourt, Christelle Harnois, Jacques Rouden, Jérôme Baudoux, Bénédicte Lepoittevin

**Affiliations:** 1 ENSICAEN, University Caen Normandie, University Rouen Normandie, INSA Rouen Normandie, CNRS, Institut CARMeN UMR 6064, 6 Bd Maréchal Juin, 14000 Caen, Francejacques.rouden@ensicaen.fr (J.R.); jerome.baudoux@ensicaen.fr (J.B.); 2 ICMMO, UMR CNRS 8182, University Paris-Saclay, 91405 Orsay, France; 3 Laboratoire de Cristallographie et Sciences des Matériaux (CRISMAT), Normandy University, UMR 6508, ENSICAEN, UNICAEN, CNRS, 6 Bd Maréchal Juin, 14000 Caen, France; arnaud.fouchet@ensicaen.fr (A.F.); christelle.harnois@ensicaen.fr (C.H.)

**Keywords:** XPS, zwitterionic polymers, surface, grafting, zirconia

## Abstract

Colonization of surfaces by bacteria followed by biofilm formation is a cause of wound infections associated with the use of medical devices as stents, catheters, implants, etc. For prevention of such infections, the preparation of surfaces with antifouling, anti-adhesive and antibacterial properties is of great interest. In this context, four zwitterionic (styrenic or methacrylic) monomers bearing a pyridinium, imidazolium or ammonium cationic group linked to a sulfonate anionic group were chosen and polymerized on ceramic for implant technology. Zwitterionic polymers were successfully grafted onto zirconia pellets through surface-initiated radical polymerization with blue-light photoactivation (“grafting from”). Wettability measurements showed the formation of hydrophilic surfaces with water contact angles in the range of 35–40°. Detailed X-ray photoelectron spectroscopy analysis revealed a surface where the zirconia pellets exhibited zwitterionic polymer brushes with high coverage. The core-level spectra of C1s, N1s and S2p were separated into many components, allowing their attribution to the different atoms in the monomer unit and confirming that zwitterionic polymers were successfully grafted from zirconia surfaces.

## 1. Introduction

In the last decade, zwitterionic polymers have attracted attention due to their unique molecular structures, in which positive and negative charges are associated within the same repeat unit, giving access to many applications, including drug delivery in the biomedical field [1], lubrication, wastewater treatment, sensor and antifouling coating, etc. [2,3]. Recently, cationic polymers with quaternary ammonium or imidazolium groups have shown promising ability to bind to bacteria and penetrate the membrane, leading to disruption of the cytoplasmic membrane and inhibition of bacterial growth [4]. In this regard, grafting zwitterionic polymers onto surfaces presents a challenge to achieving the antibacterial and low-fouling properties of the material. To this end, modifying the intrinsic properties of glass, ceramics or metals through surface-initiated graft polymerization constitutes an effective approach to preventing biofilm formation. In this field, different studies have reported the use of “grafting from” polymerization of zwitterionic monomers to prepare polymer brushes tethered on a surface for biomedical application. As a recent and illustrative example, Maison et al. described a new styrenic monomer combining a quaternary ammonium group with a sulfobetaine group. After its surface-initiated polymerization on a titanium or polyethylene surface, strong antibacterial activity associated with low-fouling properties was observed against Gram-positive and Gram-negative bacteria [5]. An overview of zwitterionic polymers and their use for biomaterials was recently presented by Q. Li et al. [6].

**Scheme 1:**
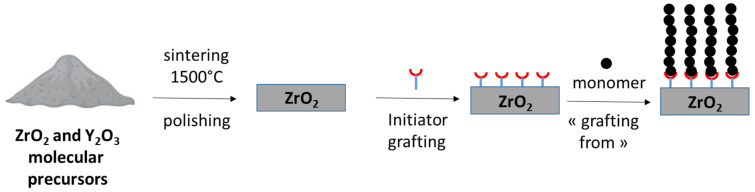
Three-step procedure for zirconia surface functionalization with polymer grafting.

In recent years, our group has developed different methodologies for the grafting of polymers on zirconia pellets with the aim of preparing biomaterial with antibacterial properties for implant technology [7,8]. We successfully showed antibacterial activity against *Staphylococcus aureus* and *Pseudomonas aeruginosa* strains after grafting imidazolium-type poly(ionic liquid) on a zirconia surface [9]. In our studies, the general scheme for the preparation of functional zirconia pellets is based on the three steps schematically presented below (Scheme 1). First, zirconia pellets are obtained with conventional ceramic technology using stabilized ZrO_2_ with 3 molar % of Y_2_O_3_. Then, a radical polymerization initiator is grafted on zirconia surfaces using a bifunctional molecule previously designed and synthesized in our laboratory [7,8]. In this step, catechol (dopamine co-deposition) [7] or phosphonic acid [8] can act as an anchoring site combined with *N*,*N*-dimethyl aromatic amine for photoinitiation activation [10]. The final step is the radical polymerization of the different monomers with a vinyl unit (styrenic or methacrylic) through light activation. In the present study, with the aim of obtaining antibacterial surfaces, we extend our methodology to the grafting of zwitterionic polymers using monomers bearing imidazolium, ammonium or pyridinium cationic groups associated with a sulfonate anionic group. To the best of our knowledge, this work describes the first example of grafting such polymers onto zirconia surfaces for biomaterial applications.

To obtain a fine characterization of the grafted surfaces, we focus our study on the use of X-ray photoelectron spectroscopy (XPS). Indeed, XPS is a reliable technique for the characterization of polymer films or surfaces grafted with polymer chains [11]. This technique allows us to understand the chemical environment of elements present on the surface at a probing depth of several nanometers, ranging from 3 to 10 nm. It also allows them to evaluate their atomic concentrations in a semi-quantitative manner (with a precision ranging from 5 to 20%). It is particularly useful in the case of zwitterionic polymers, as the shift in binding energy (BE) induced by the presence of an electrical charge on an atom (a shift toward higher BEs indicates positive charge; a shift toward lower BEs indicates negative charge) is significant. There is a characteristic BE associated with each element. In a general manner, an advantage of the XPS technique is the possibility of identifying the type of bonds between atoms and their neighbor, with variations in electron density signifying a change in their chemical environment and therefore a shift in BE. When an element, such as carbon for example, has different chemical environments, its core-level spectrum has a more complex structure, which is an addition caused by the independent contributions coming from the different chemical states of carbon. It was previously shown that XPS technology is very well suited for characterizing zwitterionic polymers grafted on surfaces, (nano)particles and membranes [12,13,14].

## 2. Experimental Section

### 2.1. Materials

All starting reagents and solvents were commercially available and used without additional purification. (±)-Camphorquinone (97%) was purchased from Merck (Rahway, NJ, USA). The synthesis of zwitterionic monomers was previously described [15,16]. Photopolymerization reactions were performed in 20 mL screw vials with 24 mm clear glass from Interchim (CK1540), using an Evolu Chem Photo RedOx Box Hepato Chem and an LED lamp (450–455 nm) purchased from Interchim (Montluçon, France).

### 2.2. Preparation of Ceramic Pellets

The Y-TZP (yttria tetragonal zirconia polycrystal) samples were prepared from a Tosoh TZ-3YB-E zirconia ZrO_2_ powder with 3 mol% Y_2_O_3_ (the particle size diameter is equal to 40 nm). A quantity of 1.5 g of the powder was introduced into a 1.6 cm diameter mold and pressed at 100 MPa. The Y-TZP disks were placed in an oven and sintered at 1500 °C for 2 h. The heating and cooling ramps were 150 °C/h. After cooling to room temperature, we obtained the samples (zirconia disks with a diameter of 13 mm and a thickness equal to 3 mm). Then, Y-TZP disks were polished in two steps: i/mechanically, with abrasive papers (220-grit, 500-grit and 1200-grit) in the presence of water, and ii/with Largo, DAC and NAP abrasive papers and diamond solutions to obtain Y-TZP disks. Finally, the Y-TZP were cleaned ultrasonically in acetone and then dried for a few minutes under an argon atmosphere. Before chemical grafting, the Y-TZP disks (diameter: 13 mm and thickness: 3 mm) were cleaned and activated by freshly prepared “piranha” solution (using 98% H_2_SO_4_ and 30% wt H_2_O_2_ in H_2_O; 70/30 *v*/*v*) for 1 h at room temperature and rinsed thoroughly with deionized water until neutral pH [7].

### 2.3. Grafting of Initiator Followed by Polymerizations

The photoinitiator (10 mg) and dopamine hydrochloride (20 mg) were solubilized in a mixture of methanol and water (1/1 *v*/*v*, 10 mL) at a pH equal to 9 (obtained with sodium hydroxide). The zirconia disks were immersed in the solution for 48 h at room temperature and rinsed thoroughly with deionized water and methanol. The zirconia disks with the grafted initiator were dried under argon and stored under vacuum before polymerization [7].

Then, a solution (H_2_O, 6 mL and CH_3_OH, 3 mL) containing zwitterionic monomer **M1**–**M4** (3 mmol), NaCl (0.35 g, 6.0 × 10^−3^ mol) and camphorquinone (0.036 g, 0.22 × 10^−3^ mol) was added under an inert atmosphere (argon). The reaction medium was irradiated using a photoreactor, which was equipped with a lamp emitting λ = 465 nm at room temperature. The functionalized ceramic surfaces were removed from the solution after two hours of polymerization and carefully washed several times with a water/methanol (2/1) mixture and aqueous NaCl solution (5 wt%), followed by water alone (to remove monomer and ungrafted polymer). Then, zirconia substrates were dried under a vacuum.

### 2.4. Characterizations

X-ray photoelectron spectroscopy (XPS) analyses were conducted using a K-Alpha spectrometer (ThermoFisher, Waltham, MA, USA) equipped with a monochromated Al Kα X-ray source (1486.6 eV). All measurements employed a 400 μm spot size, corresponding to an irradiated area of approximately 1 mm^2^. The hemispherical analyzer operated in Constant Analyzer Energy (CAE) mode, with the acquisition parameters of 200 eV pass energy and a 1 eV step for survey spectra and 50 eV pass energy with a 0.1 eV step for high-resolution narrow scan spectra. Charge neutralization was achieved using a dual-beam flood gun. Base pressure in the analysis chamber was maintained at approximately 2 × 10^−9^ mbar. Spectral processing was performed using Avantage software (https://www.thermofisher.com/fr/fr/home.html, accessed on 1 January 2025) with a peak-fitting routine incorporating Shirley background subtraction and symmetrical 70–30% mixed Gaussian–Lorentzian line shapes. Atomic percentages were calculated through the normalization of peak areas using Scofield sensitivity factors. During fitting procedures, components added to core-level spectra were constrained to identical line shapes and a similar full width at half maximum (FWHM). Component selection was based on molecular chemical structure and established chemical shift data from the literature and databases. Additional binding energy and area ratio constraints were implemented between components. Fit quality was verified by comparing normalized areas of corresponding components across all core-level spectra, with precision within expected XPS limits (5–15%). The binding energy scale was calibrated against neutral carbon at 285 eV, with a precision of ±0.2 eV. Surface homogeneity of the grafted layer was assessed by analyzing four distinct surface regions.

Contact angle measurements were carried out with the Young–Laplace method, using a DSA30 Krüss goniometer (Krüss GmhH, 91550 Paris—Orly, France). High-purity water from Millipore (Molsheim, France) (milli-Q^®^ water purification system) was used. The static contact angle was measured within 5 s of placing the drop (1 × 10^−6^ L) on the surfaces, and an average of five measurements was reported.

The topography and the roughness of the zirconia substrates were determined using a Pico SPM-LE AFM (Atomic Force Microscopy) from Molecular Imaging in tapping mode (Scientec, 91940 Les Ulis, France).

## 3. Results and Discussion

### 3.1. Grafting of Zwitterionic Polymers on Zirconia Pellets

**Figure 1:**
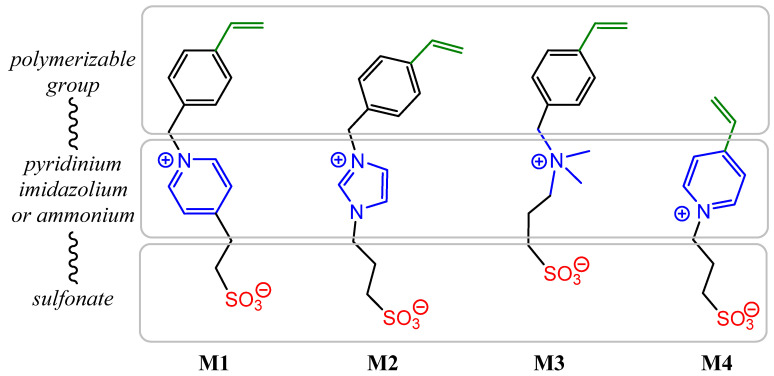
Chemical structures of zwitterionic monomers denoted **M1** to **M4** used in this study.

In agreement with our previous results, three monomers bearing a polymerizable 4-vinylbenzyl group (**M1**, **M2** and **M3**) and another vinylpyridine monomer (**M4**) were chosen with the aim of using radical polymerization with photochemical activation [7]. These monomers were prepared on a large scale in one or two steps according to previously reported procedures [15,16,17]. Their chemical structures were given on Figure 1.

Thus, the final behavior of these zwitterionic grafted polymers bearing a heterocycle (pyridinium or imidazolium) or more flexible acyclic ammonium, associated with an anionic sulfonate group, was analyzed using X-ray photoelectron spectroscopy.

**Scheme 2:**
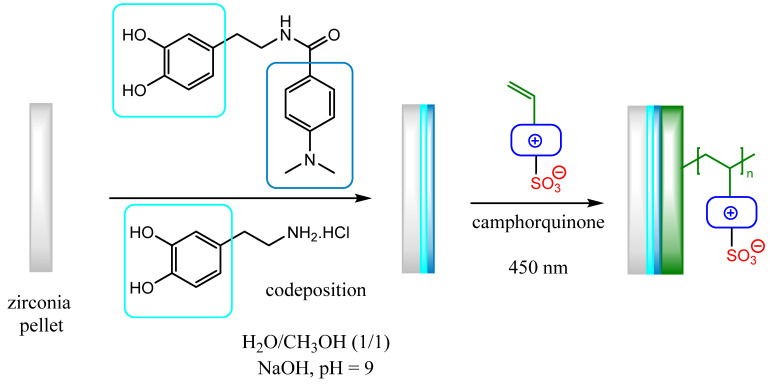
Grafting of initiator on zirconia pellets followed by photopolymerization of zwitterionic monomers **M1**–**M4** with photochemical activation. The first step is described in our previous publication [7].

Zirconia ceramic pellets (Y-TZP for tetragonal zirconia polycrystal) were prepared by sintering from a mixture of ZrO_2_ and Y_2_O_3_ powders. A photoinitiator combining a 4-dimethylaminophenyl group (radical polymerization) with a catechol group was prepared and grafted on zirconia pellets by co-deposition with dopamine in basic conditions according to previously reported conditions in our group [7]. Then, in the last step, radical photopolymerizations of the four monomers were performed in water/methanol solution with camphorquinone, a visible light photoinitiator (activation at 465 nm), at room temperature (Scheme 2).

After photopolymerization, the zirconia pellets were thoroughly washed with a mixture of polar and protic solvents to remove undesirable species such as residual monomer, camphorquinone, and ungrafted polymer. Measurements of water contact angles allow the determination of the effect of polymerization on surface wettability. After grafting the photoinitiator through the co-deposition of dopamine, the reference contact angle with water was 61°. After polymerization with the different zwitterionic monomers **M1** to **M4**, the contact angles with water were between 35 and 40°. This test indicated an increase in the hydrophilic character and thus confirmed the covering of the zirconia surface by a polymer carrying zwitterionic groups. The exact value for the four zwitterionic polymers **P1**–**P4** is reported in Table 1. These values are in good agreement with those reported in the literature for grafted zwitterionic polymers [15,16]. For example, a study reporting the surface-initiated atom transfer radical polymerization of monomer **M3** from cellulose membrane shows a water contact angle decrease from 73 to 36° according to the reaction time [18].

### 3.2. Analysis of Functionalized Surfaces Using XPS and AFM

**Figure 2:**
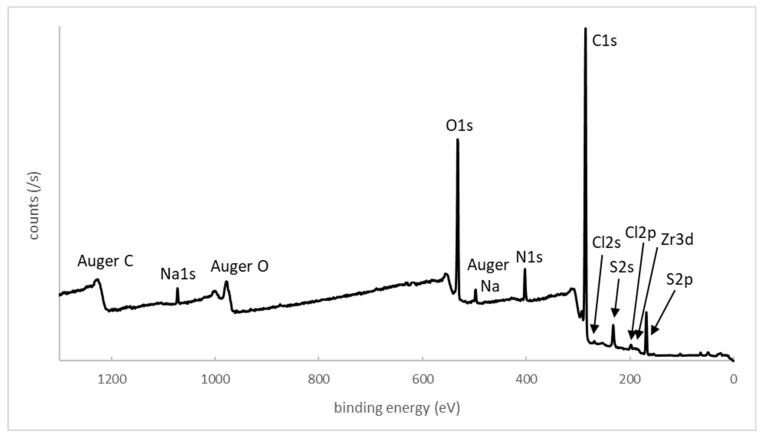
XPS characterization of Y-TZP surface grafted with zwitterionic polymer **P1**: a survey.

We focused on the XPS study of the zirconia surface obtained after photopolymerization of monomer **M1** bearing a pyridinium cationic group. The XPS wide-scan spectrum is presented in Figure 2. Calculated and experimental atomic percentages are detailed in Table 2. After polymerization, the atomic composition of carbon was significant (74.1% measured compared to 77.2% theoretically calculated from the monomer unit C_16_H_17_NO_3_S), and the oxygen composition was equal to 15.7%, in good agreement with the theoretical value equal to 13.6%. The XPS spectrum also demonstrated the presence of additional peaks associated with the zwitterionic groups. Thus, the experimental atomic percentage of sulfur (from the sulfonate group) was determined to be 5.3%, close to the theoretical value of 4.5%. The experimental atomic percentage of nitrogen (pyridinium group) was equal to 3.9%, also in good agreement with the theoretical value of 4.5%. Some residual peaks coming from the zirconia sublayer (Zr3d) and NaCl (Na1s and Cl2p) used for monomer solubilizing during polymerization were present.

**Figure 3:**
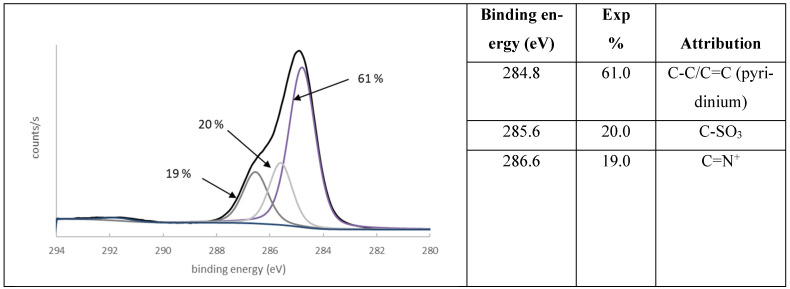
Core-level spectrum of C1s of polymer **P1** and atomic attribution of main components.

Various pyridinium-based ionic liquids have previously been investigated using XPS and described in the literature. The core-level C1s spectrum of this aromatic ring was generally fitted with two or three peaks due to different chemical environments resulting from a delocalization of the cationic charge [19,20]. The component at 286.6 eV (FWHM = 1.1 eV) was assigned to carbon bonded to nitrogen in the pyridinium group. The component at 285.6 eV (FWHM = 1.0 eV) was attributed to the carbon atom directly bonded to the sulfonate group [21]. The main peak at 284.8 eV (FWHM = 1.0 eV) corresponded to the remaining aliphatic and aromatic carbons (Figure 3).

In addition, a π-π∗ shake-up satellite peak centered at 291.7 eV was due to the interaction of photoelectrons with the π levels, generating π-π∗ transitions. This way, the photoelectrons lose some of their kinetic energy and appear at higher BEs in the spectrum.

**Figure 4:**
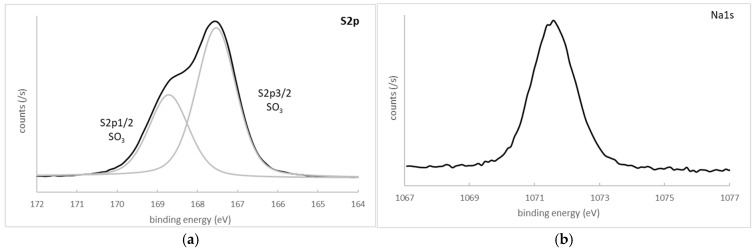
Core-level spectra of decomposition and curve-fitted spectra of S2p and Na1s of polymer **P1**. (**a**) S2p; (**b**) Na1s.

The S2p core-level spectrum contained only one spin–orbit doublet, with its S2p_3/2_ component located at 167.5 eV and a FWHM of 1.2 eV, which was attributed to a sulfonate functional group [20]. The spectrum is presented in Figure 4.

**Figure 5:**
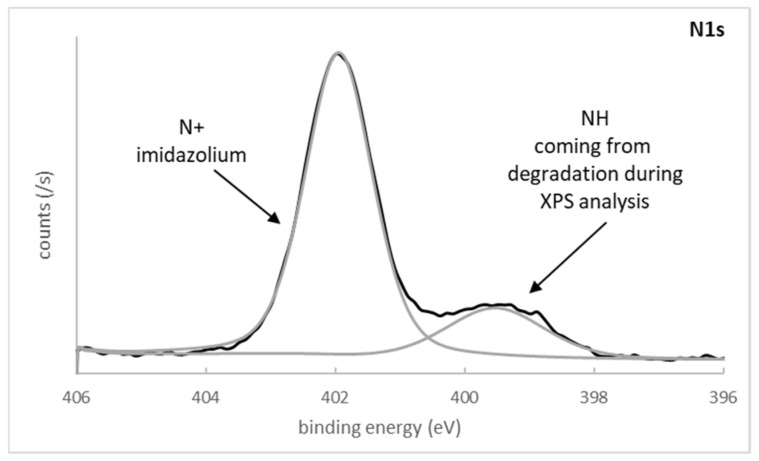
High-resolution spectrum and decomposition of N1s after monomer **M1** photopolymerization.

The core-level spectrum of N1s can be separated into two components (Figure 5) with a main peak at 402.0 eV (FWHM = 1.2 eV), attributed to the single heterocyclic nitrogen (charged) present within the polymer **P1**. This binding energy was in good agreement with literature values for polymers bearing a pyridinium ring [22]. Despite this observation, a second minor component centered at 399.5 eV (FWHM = 1.8 eV) was clearly highlighted. A possible cause could be nitrogen residues from the initiator molecule (amine), but we assume that the thickness of the grafted polymer was high enough to mask the zirconia underlayer.

Another more reasonable possibility would be time-dependent degradation of the polymer layer with the XPS spectrometer, either due to the vacuum, the effects of X-ray exposure, or both. Indeed, Zuilhof et al. hypothesized that for grafted polymers bearing a quaternary ammonium group, the time between sample preparation and XPS analysis has an effect on the outcome [23].

A Na1s peak of a small intensity coming from the presence of residual NaCl (used for monomer and polymer solubility during photopolymerization and incompletely removed by the washings) was measured at 1071.6 eV (FWHM = 1.5 eV) (Figure 4). Indeed, zwitterionic species have a low solubility in pure water, and their solubility increases with the addition of salts [24]. After reaction, an efficient removal of these atoms is quite difficult in the presence of zwitterionic units.

As a conclusion, the results of XPS analysis proved the success of **M1** monomer photopolymerization, with the formation of a homogeneous polymeric film on the surface of the Y-doped zirconia containing the initiator. Complete characterization of the other grafted polymers, **P2**–**P4**, is currently under investigation using a combination of XPS and ellipsometry techniques.

**Figure 6:**
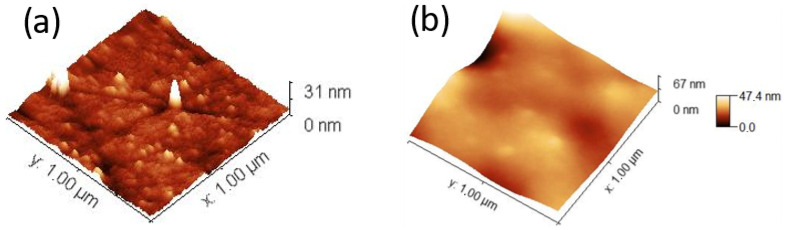
Three-dimensional AFM height image after polishing (**a**) and after monomer M1 photopolymerization (**b**).

The topography of the zirconia surface just after polishing and after monomer **M1** photopolymerization, investigated using AFM, is shown in Figure 6a,b, respectively. The surface morphology of this surface grafted with zwitterionic polymer seems uniform and does not present the grains and polishing marks observed just after polishing, in agreement with our results obtained after methyl methacrylate polymerization [7].

## 4. Conclusions

In this study, we reported the chemical modification of zirconia surfaces through the photopolymerization of zwitterionic monomers bearing an imidazolium, pyridinium or ammonium cation and a sulfonate anion. A series of four zwitterionic monomers (bearing imidazolium, ammonium or pyridinium cationic groups and a sulfonate anionic group) were successfully prepared on large scale with a styrene moiety or a vinyl-pyridinium polymerizable group. After grafting a catechol molecule with a *N*,*N*-dimethylamine group onto the surface, radical photopolymerization of the monomers was carried out using the “grafting from” strategy, with blue-light activation in the presence of camphorquinone. Static water contact angle measurements showed the production of hydrophilic surfaces with values between 35 and 40°, attesting to the success of the grafting. Subsequently, an in-depth characterization of these modified surfaces was carried out using XPS analysis to precisely point out the zwitterionic pattern with the presence of the two charged species, namely nitrogen and sulfur, in a predominantly carbon-based environment. This technique allows us to undoubtedly confirm the presence of ionic groups coming from zwitterionic polymer grafts. In the next study, the antibacterial activity of these zirconia substrates grafted with zwitterionic polymers will be evaluated against *Staphylococcus aureus* and *Pseudomonas aeruginosa* strains.

## Figures and Tables

**Table 1:** Zwitterionic polymer chemical structures and water contact angle of the zirconia surface after photopolymerization.

Monomer	M1	M2	M3	M4
Polymer	P1	P2	P3	P4
**Polymer chemical structure**	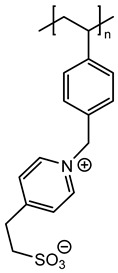	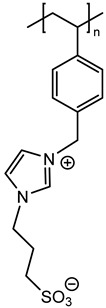	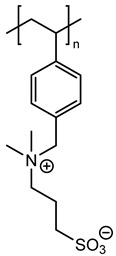	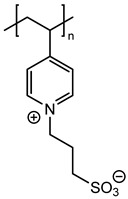
**Water contact angle (°)**	38 ± 2	39 ± 3	41 ± 3	36 ± 2

**Table 2:** Theoretical and experimental atomic percentages determined using XPS for polymer **P1**.

	**Atomic %** **Theoretical** ^a^	**Atomic %** **Experimental**	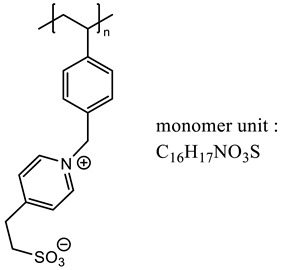
**C1s**	77.2	74.1	
**O1s**	13.6	15.7	
**S2p**	4.5	5.3	
**N1s**	4.5	3.9	
**Zr3d**	-	<1	
**Na1s**	-	<1	
**Cl2p**	-	<1	

^a^ calculated from the monomer unit formula C_16_H_17_NO_3_S.

## Data Availability

The original contributions presented in this study are included in the article. Further inquiries can be directed to the corresponding authors.
